# Population and genomic analysis of the genus *Halorubrum*

**DOI:** 10.3389/fmicb.2014.00140

**Published:** 2014-04-11

**Authors:** Matthew S. Fullmer, Shannon M. Soucy, Kristen S. Swithers, Andrea M. Makkay, Ryan Wheeler, Antonio Ventosa, J. Peter Gogarten, R. Thane Papke

**Affiliations:** ^1^Department of Molecular and Cell Biology, University of ConnecticutStorrs, CT, USA; ^2^Department of Cell Biology, Yale School of Medicine, Yale UniversityNew Haven, CT, USA; ^3^Department of Microbiology and Parasitology, University of SevilleSeville, Spain

**Keywords:** Halobacteria, Multilocus Sequence Analysis (MLSA), Average Nucleotide Identity (ANI), intein, CRISPR

## Abstract

The Halobacteria are known to engage in frequent gene transfer and homologous recombination. For stably diverged lineages to persist some checks on the rate of between lineage recombination must exist. We surveyed a group of isolates from the Aran-Bidgol endorheic lake in Iran and sequenced a selection of them. Multilocus Sequence Analysis (MLSA) and Average Nucleotide Identity (ANI) revealed multiple clusters (phylogroups) of organisms present in the lake. Patterns of intein and Clustered Regularly Interspaced Short Palindromic Repeats (CRISPRs) presence/absence and their sequence similarity, GC usage along with the ANI and the identities of the genes used in the MLSA revealed that two of these clusters share an exchange bias toward others in their phylogroup while showing reduced rates of exchange with other organisms in the environment. However, a third cluster, composed in part of named species from other areas of central Asia, displayed many indications of variability in exchange partners, from within the lake as well as outside the lake. We conclude that barriers to gene exchange exist between the two purely Aran-Bidgol phylogroups, and that the third cluster with members from other regions is not a single population and likely reflects an amalgamation of several populations.

## Introduction

Besides an obligate requirement for high concentrations of NaCl, a unifying trait of Halobacteria (often referred to colloquially as the haloarchaea)—a class within the archaeal phylum Euryarchaeota, is their propensity for horizontal gene transfer (HGT) (Legault et al., 2006; Rhodes et al., 2011; Nelson-Sathi et al., 2012; Williams et al., 2012). Although HGT occurs continuously, events that provide an adaptive advantage and are maintained in modern lineages can be detected. For instance, HGTs from bacterial lineages into the Halobacteria occurred before their last common ancestor and brought respiration and nutrient transport genes that transformed them from a methanogen to their current aerobic heterotrophic state (Nelson-Sathi et al., 2012). Other examples including rhodopsins (Sharma et al., 2006), tRNA synthetases (Andam et al., 2012), 16S rRNA genes (Boucher et al., 2004), membrane proteins (Cuadros-Orellana et al., 2007), and genes allowing the assembly of novel pathways (Khomyakova et al., 2011) have been reported for this group and reflect the adaptive benefit of acquiring these genes.

HGT into the Halobacteria has profoundly impacted their evolution; however, understanding this contribution is only part of their evolutionary picture. The study of recombination frequency among this class has been utilized to address population genetics questions that address whether they are clonal (i.e., linked alleles at different loci) or “sexual” in the sense that alleles at different loci are randomly associated. Several studies have addressed those questions by assessing the impact of frequent HGT on Halobacteria. Homologous replacement of loci was inferred within and between phylogenetic clusters (phylogroups) using Multilocus Sequence Analysis (MLSA) on closely related strains (Papke et al., 2004) and comparative analyses of genomes (Williams et al., 2012). Within phylogroups where genetic diversity was less than one percent divergent for protein coding genes, alleles at different loci were randomly associated whereas between phylogroups they were not (Papke et al., 2007) indicating haloarchaea are highly sexual. Measurements of frequency across the breadth of halobacterial diversity indicates no absolute barrier to homologous recombination; rather between relatives, there is a log-linear decay in recombination frequency relative to phylogenetic distance (Williams et al., 2012).

Laboratory experiments also support these results. Mating experiments measuring the rate of recombination using *Haloferax (Hfx) volcanii* and *Hfx. mediterranei* auxotrophs demonstrated the degree of genetic isolation between species was much lower than expected. The observed rate of exchange between species suggested that given an opportunity over time these species would homogenize, indicating strong barriers to recombination would have to exist for speciation to occur, and for lineages to be maintained (Naor et al., 2012). Further, mating experiments demonstrated that enormous genomic fragments (i.e., 300–500 kb, ~18% of the chromosome size) could be exchanged in a single event (Naor et al., 2012). Similar large fragment exchange events were recently observed in natural isolates from Deep Lake (Antarctic hypersaline lake): Distantly related strains (<75% average nucleotide identity) shared up to 35 kb with nearly 100% sequence identity (DeMaere et al., 2013).

The Halobacteria have clearly been shaped by gene transfer and are actively engaged in substantial genetic exchange. However, little is known about genomic diversity within populations, and the impact of gene flow is unknown at these scales. In this study we report the intra and inter population sequence diversity of *Halorubrum* spp. strains cultivated from the same location and compare them to the genomic diversity of type strains from the same genus. Our results lead to insights on the genomic diversity that comprises haloarchaeal species.

## Methods

### Growth conditions and DNA extraction

*Halorubrum* spp. cultures were grown in Hv-YPC medium (Allers et al., 2004) at 37°C with agitation. DNA from Halobacteria was isolated as described in the Halohandbook (Dyall-Smith, 2009). Briefly, stationary-phase cells were pelleted at 10,000 × *g*, supernatant was removed and the cells were lysed in distilled water. An equal volume of phenol was added, and the mixture was incubated at 65°C for 1 h prior to centrifugation to separate the phases. The aqueous phase was reserved and phenol extraction was repeated without incubation, and followed with a phenol/chloroform/iso-amyl alcohol (25:24:1) extraction. The DNA was precipitated with ethanol, washed, and re-suspended in TE (10 mM tris, pH 8.0, 1 mM EDTA).

### Multilocus sequence analysis (MLSA)

Five housekeeping genes were amplified using PCR. The loci were *atpB*, *ef-2*, *glnA*, *ppsA*, and *rpoB* and the primers used for each locus are listed in Table 1. To more efficiently sequence PCR products, an 18 bp M13 sequencing primer was added to the 5' end of each degenerate primer (Table 1). Each PCR reaction was 20 μ l in volume. The PCR reaction was run on a Mastercycler Ep Thermocycler (Eppendorf) using the following PCR cycle protocol: 30 s initial denaturation at 98°C, followed by 40 cycles of 30 s at 98°C, 5 s at the annealing temperature for each set of primers and 15 s at 72°C. Final elongation occurred at 72°C for 1 min. Table 2 provides a detailed list of reagents and the PCR mixtures for each amplified locus. The PCR products were separated by gel electrophoresis with agarose (1%). Gels were stained with ethidium bromide. An exACTGene mid-range plus DNA ladder (Fisher Scientific International Inc.) was used to estimate the size of the amplicons, which were purified using Wizard SV gel and PCR cleanup system (Promega). The purified amplicons were sequenced by Genewiz Inc. using Sanger sequencing technology.

**Table 1:** **Degenerate primers used to PCR amplify and sequence the genes for MLSA**.

**MLSA primer sequence 5'–3'**		
**Locus**	**Forward**	**Reverse**
atpB	tgt aaa acg acg gcc agt aac ggt gag scv ats aac cc	cag gaa aca gct atg act tca ggt cvg trt aca tgt a
ef-2	tgt aaa acg acg gcc agt atc cgc gct bta yaa stg g	cag gaa aca gct atg act ggt cga tgg wyt cga ahg g
glnA	tgt aaa acg acg gcc agt cag gta cgg gtt aca sga cgg	cag gaa aca gct atg acc ctc gcs ccg aar gac ctc gc
ppsA	tgt aaa acg acg gcc agt ccg cgg tar ccv agc atc gg	cag gaa aca gct atg aca tcg tca ccg acg arg gyg g
rpoB	tgt aaa acg acg gcc agt tcg aag agc cgg acg aca tgg	cag gaa aca gct atg acc ggt cag cac ctg bac cgg ncc

**Table 2:** **PCR conditions for each locus**.

	**atpB**	**ef-2**	**glnA**	**ppsA**	**rpoB**
Water (μl)	11.6	8.2	11.8	7.9	11.9
5× phire reaction buffer (μl)	4.0	4.0	4.0	4.0	4.0
DMSO (μl)	0.6	0	0.4	0.6	0.6
Acetamide (25%, μl)	0	4.0	0	4.0	0
dNTP mix (10 mM, μl)	0.4	0.4	0.4	0.4	0.4
Forward primer (10 mM, μl)	1.0	1.0	1.0	1.0	1.0
Reverse primer (10 mM, μl)	1.0	1.0	1.0	1.0	1.0
Phire II DNA polymerase (μl)	0.4	0.4	0.4	0.4	0.4
Template DNA (20 ng/μl, μl)	1.0	1.0	1.0	0.7	0.7
Annealing temperature (°C)	60.0	61.0	69.6	66.0	63.7

### Genome sequencing

DNA purity was analyzed with a Nanodrop spectrophotometer, was quantified using a Qubit fluorometer (Invitrogen) and then prepared for sequencing using the Illumina Nextera XT sample preparation kit as described by the manufacturer. Fragmented and amplified libraries were either normalized using the normalization beads and protocol supplied with the kit, or manually as described in protocols for the Illumina Nextera kit. Libraries were loaded onto 500 cycle MiSeq reagent kits with a 5% spike-in PhiX control, and sequenced using an Illumina MiSeq benchtop sequencer. The genomes to be sequenced were selected based upon the results of the initial PCR MLSA data analysis (see Results).

### Genome assembly

Type strain genomes were obtained from the NCBI ftp repository. *Halorubrum lacusprofundi* and the non-*Halorubrum* genomes (*Haloarcula marismortui* ATCC 43049 and *Har. hispanica* ATCC 33960 as well as *Haloferax volcanii* DS2 and *Hfx. mediterranei* ATCC 33500) are completed projects. The other *Halorubrum* genomes are drafts, also obtained from the NCBI ftp repository. New draft genomes were sequenced using an Illumina MiSeq platform. Assembly on strain Ga2p was carried out using the ngopt A5 pipeline(Tritt et al., 2012) while all others were assembled via the CLC Genomics Workbench 6.0.5 suite with a trim and merge workflow with scaffolding enabled.

To ensure equal gene calling across the genomes all genomes, including the 19 draft and completed *Halorubrum*, *Haloferax,* and *Haloarcula* genomes available on the NCBI ftp site as of June 2013, were reannotated using the rapid annotation using subsystem technology (RAST) server (Aziz et al., 2008). Assembled contigs were reconstructed from the RAST-generated genbank files for all genomes using the seqret application of the emboss package (Rice et al., 2000).

### Phylogenetic methodology

Top scoring BLASTn hits for each MLSA target gene (*atpB*, *ef-2*, *glnA*, *ppsA*, and *rpoB*) in each genome were identified. Multiple-sequence alignments (MSAs) were generated by translating the genes to protein sequences in SeaView (Gouy et al., 2010), aligning the proteins using MUSCLE (v.3.8.31) (Edgar, 2004) and then reverting back to the nucleotide sequences. In-house scripts created a concatenated alignment of all five genes. The best model of evolution was determined by calculating the Akaike Information Criterion with correction for small sample size (AICc) in jModelTest 2.1.4 (Guindon et al., 2010; Darriba et al., 2012). The best-fitting model was GTR + Gamma estimation + Invariable site estimation. A maximum likelihood (ML) phylogeny was generated from the concatenated MSA and individual gene phylogenies from the individual gene MSAs using PhyML (v3.0_360-500M)(Guindon et al., 2010). PhyML parameters consisted of GTR model, estimated p-invar, 4 substitution rate categories, estimated gamma distribution, subtree pruning, and regrafting enabled with 100 bootstrap replicates.

### Pairwise sequence identity calculation

Calculation of pairwise identities was carried out using Clustal Omega on the EMBL-EBI webserver (http://www.ebi.ac.uk/Tools/msa/clustalo/). The alignments were uploaded and percent identity matrices calculated (Sievers et al., 2011).

### Intein methodology

To retrieve haloarchaeal intein sequences Position-Specific Scoring Matrices (PSSMs) were created using the collection of all inteins from InBase, the Intein database, and registry (Perler, 2002). A custom database was created with all inteins, and each intein was used as a seed to create a PSSM using the custom database. These PSSMs were then used as a seed for PSI-BLAST (Altschul et al., 1997) against each of the halobacterial genomes available from NCBI. A size exclusion step was then performed to remove false positives. Inteins were then aligned using MUSCLE (Edgar, 2004) with default parameters in the SeaView version 4.0 software package (Gouy et al., 2010). Insertions, which passed the size exclusion step but did not contain splicing domains, were filtered out and the previous steps were repeated using the resulting dataset on this study's dataset. Once the collection of haloarchaeal inteins was complete, sequences were re-aligned using SATé v2.2.2 (Liu et al., 2012) to generate a final alignment.

### Intein phylogenetic methodology

Intein protein sequences were retrieved using in house scripts. Each intein allele was aligned separately using MUSCLE (v.3.8.31) (Edgar, 2004). In-house scripts created a concatenated alignment from the allele alignments. ProtTest v3.4 (Darriba et al., 2011) evaluated the protein sequences for an optimal model using the AICc and returned WAG_I+G+F. A presence-absence matrix of zeros and ones was amended to each taxon's alignment data. The presence-absence data allows for grouping of taxa by sharing or lacking an allele. This complements the protein data, and allows the resolution of taxa with few inteins from those lacking them entirely or possessing many. To accommodate the two different formats of data simultaneously MrBayes v3.2.2 (Ronquist and Huelsenbeck, 2003; Ronquist et al., 2012) was employed for the phylogenetic reconstruction.

### Average nucleotide identity/tetramer analysis

JSpecies1.2.1 (Richter and Rosselló-Móra, 2009) was used to analyze the genomes for Average Nucleotide Identity (ANI) and tetramer frequency patterns. As the relationships of interest for this study are within the same genus only the nucmer and tetra algorithms were used. The BLAST-based ANI was not used as we were primarily interested in understanding the degree of relatedness between closely related organisms, which the nucmer method is equally capable of (Richter and Rosselló-Móra, 2009). Additionally, the increased rate of drop-off between moderately divergent sequences (<90%) the nucmer method yields relative to the BLAST method (Richter and Rosselló-Móra, 2009) was useful in highlighting when organisms were dissimilar. The default settings for both algorithms were used (Richter and Rosselló-Móra, 2009).

### Codon position GC content

Complete sets of nucleotide sequences for all called ORFs were downloaded from RAST. In house scripts confirmed that all ORF calls were divisible by three and thus could be taken as in-frame. In house scripts were used to calculate the GC percentages for each codon position in each genome. Two-tailed *t*-tests were calculated using the StatsPlus software package (AnalystSoft, 2009).

### CRISPRs

Clustered Regularly Interspaced Short Palindromic Repeats (CRISPRs) presence/absence patterns were determined using the CRISPR Recognition Tool (CRT) v1.2 (Bland et al., 2007) with minimum repeat and minimum spacer parameters set to 30 nucleotides. All other parameters were the CRT defaults.

## Results

### Assembled genomes

The assembled genomes ranged in size from 2.3 to 4.2 Mb. The median assembled genome size is 3.6 Mb. The median N50 (the size of the contig where 50% of the basepairs in the assembly are part of a contig that size or larger. N75 and N90 are similar but use 75 and 90% cutoffs) was 47.5 kb with a range from 1.86 to 80.3 kb (see Table 3, for statistics on the assembled genomes). Plasmids were not identified during assembly. As such, if some isolates possess differing numbers or types of plasmids then some of the genome-to-genome size variability may be attributable to this. A list of genomes used in this study can be found in Table 4.

**Table 3:** **Assembly statistics for the genomes sequenced in this study**.

	**C191**	**C3**	**C49**	**Cb34**	**E3**	**E8**	**Ea1**	**Ea8**	**Eb13**	**Ec15**	**Fb21**	**G37**	**Ga2p**	**Ga36**	**Hd13**	**Ib24**	**LD3**	**LG1**
N75 (kb)	18.9	2.3	23.2	24.7	1.1	1.3	30.0	25.1	25.4	42.7	25.3	27.2	41.1	23.8	32.1	23.2	21.4	8.4
N50 (kb)	54.9	4.4	56.3	42.9	1.9	2.3	43.8	51.6	51.6	80.3	42.7	68.1	74.9	51.2	64.4	43.4	39.6	32.1
N25 (kb)	97.3	7.8	99.8	73.4	3.5	4.0	77.5	95.4	95.7	131.8	90.3	118.4	118.9	91.9	83.0	68.2	76.0	67.9
Minimum (kb)	0.5	0.4	0.5	0.5	0.4	0.4	0.5	0.5	0.5	0.6	0.5	0.5	0.3	0.5	0.5	0.5	0.5	0.4
Maximum (kb)	180.2	40.5	183.6	123.4	26.7	25.0	203.3	169.6	268.1	412.4	174.7	230.0	246.3	145.6	122.0	190.3	145.8	153.4
Average (kb)	16.6	2.9	22.5	23.1	1.5	1.8	24.7	22.6	23.3	44.3	20.6	25.7	40.3	21.0	27.9	19.6	17.5	4.4
Contig count	233	1165	159	145	2764	1278	159	166	156	74	176	138	83	160	137	189	213	1090
Length (Mb)	3.87	3.33	3.58	3.35	4.21	2.26	3.93	3.75	3.63	3.28	3.63	3.55	3.35	3.36	3.82	3.70	3.73	4.79
Base composition (GC%)	66.0	65.8	65.8	67.6	65.5	66.3	67.0	67.6	67.5	67.6	66.6	67.1	67.8	67.7	67.6	67.6	66.2	66.0
Number of coding sequences	3908	3379	3529	3323	4147	2187	3977	3672	3544	3245	3600	3617	3400	3382	3718	3612	3724	4615
Number of RNAs	57	37	49	54	51	31	50	49	48	47	65	48	49	47	51	48	56	69

**Table 4:** **List of genomes used in this study**.

**Organism name**	**NCBI identifier**	**Sequence source**	**Isolation site**	**Environment**	**Status**
*Haloarcula hispanica* ATCC 33960	PRJNA72475	NCBI	Alicante, Spain	Solar saltern	Complete
*Haloarcula marismortui* ATCC 43049	PRJNA57719	NCBI	Dead Sea, Israel	Saline lake/sea	Complete
*Haloferax mediterranei* ATCC 33500	PRJNA167315	NCBI	Alicante, Spain	Solar saltern	Complete
*Haloferax volcanii* DS2	PRJNA46845	NCBI	Dead Sea, Israel	Saline lake/sea	Complete
*Halorubrum* sp. T3	PRJNA199598	NCBI	Yunnan, China	Solar saltern	Draft
*Halorubrum aidingense* JCM 13560	PRJNA188616	NCBI	Xin-Jiang, China	Saline lake	Draft
*Halorubrum arcis* JCM 13916	PRJNA188617	NCBI	Xin-Jiang, China	Saline lake	Draft
*Halorubrum californiensis* DSM 19288	PRJNA188618	NCBI	California, United States	Solar saltern	Draft
*Halorubrum coriense* DSM 10284	PRJNA188619	NCBI	Geelong, Australia	Solar saltern	Draft
*Halorubrum distributum* JCM 10118	PRJNA188621	NCBI	Turkmenistan	Saline soils	Draft
*Halorubrum distributum* JCM 9100	PRJNA188620	NCBI	Turkmenistan	Saline soils	Draft
*Halorubrum hochstenium* ATCC 700873	PRJNA188622	NCBI	California, United States	Solar saltern	Draft
*Halorubrum kocurii* JCM 14978	PRJNA188615	NCBI	Inner Mongolia, China	Saline lake	Draft
*Halorubrum lacusprofundi* ATCC 49239	PRJNA58807	NCBI	Deep Lake, Antarctica	Saline lake	Complete
*Halorubrum lipolyticum* DSM 21995	PRJNA188614	NCBI	Xin-Jiang, China	Saline lake	Draft
*Halorubrum litoreum* JCM 13561	PRJNA188613	NCBI	Fujian, China	Solar saltern	Draft
*Halorubrum saccharovorum* DSM 1137	PRJNA188612	NCBI	California, United States	Solar saltern	Draft
*Halorubrum tebenquichense* DSM 14210	PRJNA188611	NCBI	Atacama, Chile	Solar saltern	Draft
*Halorubrum terrestre* JCM 10247	PRJNA188610	NCBI	Turkmenistan	Saline soils	Draft
Hrr. Cb34	PRJNA232799 (in submission)	This study	Aran-Bidgol, Iran	Saline lake	Draft
Hrr. C49	PRJNA232799 (in submission)	This study	Aran-Bidgol, Iran	Saline lake	Draft
Hrr. Ea1	PRJNA232799 (in submission)	This study	Aran-Bidgol, Iran	Saline lake	Draft
Hrr. Eb13	PRJNA232799 (in submission)	This study	Aran-Bidgol, Iran	Saline lake	Draft
Hrr. Ib24	PRJNA232799 (in submission)	This study	Aran-Bidgol, Iran	Saline lake	Draft
Hrr. Ea8	PRJNA232799 (in submission)	This study	Aran-Bidgol, Iran	Saline lake	Draft
Hrr. Hd13	PRJNA232799 (in submission)	This study	Aran-Bidgol, Iran	Saline lake	Draft
Hrr. C3	PRJNA232799 (in submission)	This study	Aran-Bidgol, Iran	Saline lake	Draft
Hrr. E8	PRJNA232799 (in submission)	This study	Aran-Bidgol, Iran	Saline lake	Draft
Hrr. E3	PRJNA232799 (in submission)	This study	Aran-Bidgol, Iran	Saline lake	Draft
Hrr. LG1	PRJNA232799 (in submission)	This study	Aran-Bidgol, Iran	Saline lake	Draft
Hrr. Fb21	PRJNA232799 (in submission)	This study	Aran-Bidgol, Iran	Saline lake	Draft
Hrr. Ga2p	PRJNA232799 (in submission)	This study	Aran-Bidgol, Iran	Saline lake	Draft
Hrr. G37	PRJNA232799 (in submission)	This study	Aran-Bidgol, Iran	Saline lake	Draft
Hrr. LD3	PRJNA232799 (in submission)	This study	Aran-Bidgol, Iran	Saline lake	Draft
Hrr. Ec15	PRJNA232799 (in submission)	This study	Aran-Bidgol, Iran	Saline lake	Draft
Hrr. Ga36	PRJNA232799 (in submission)	This study	Aran-Bidgol, Iran	Saline lake	Draft

### Phylogenetic assignment of phylogroups

**Figure 1:**
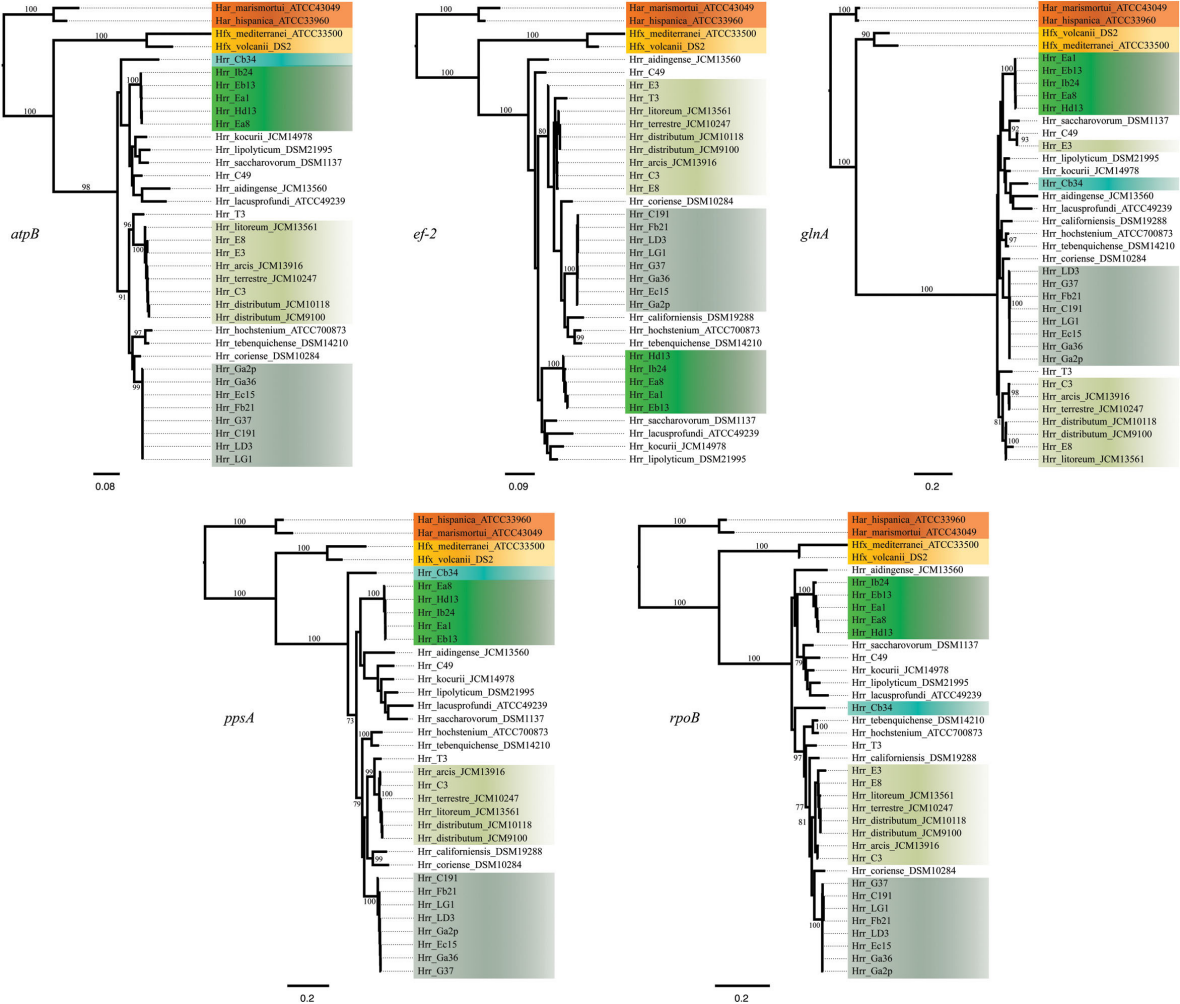
**Maximum-likelihood gene trees made from the DNA sequences of *atpB, ef-2*, *glnA*, *ppsA*, and *rpoB***. Support values on branches are bootstrap replicates. Bootstrap values below 70 are not displayed.

**Figure 2:**
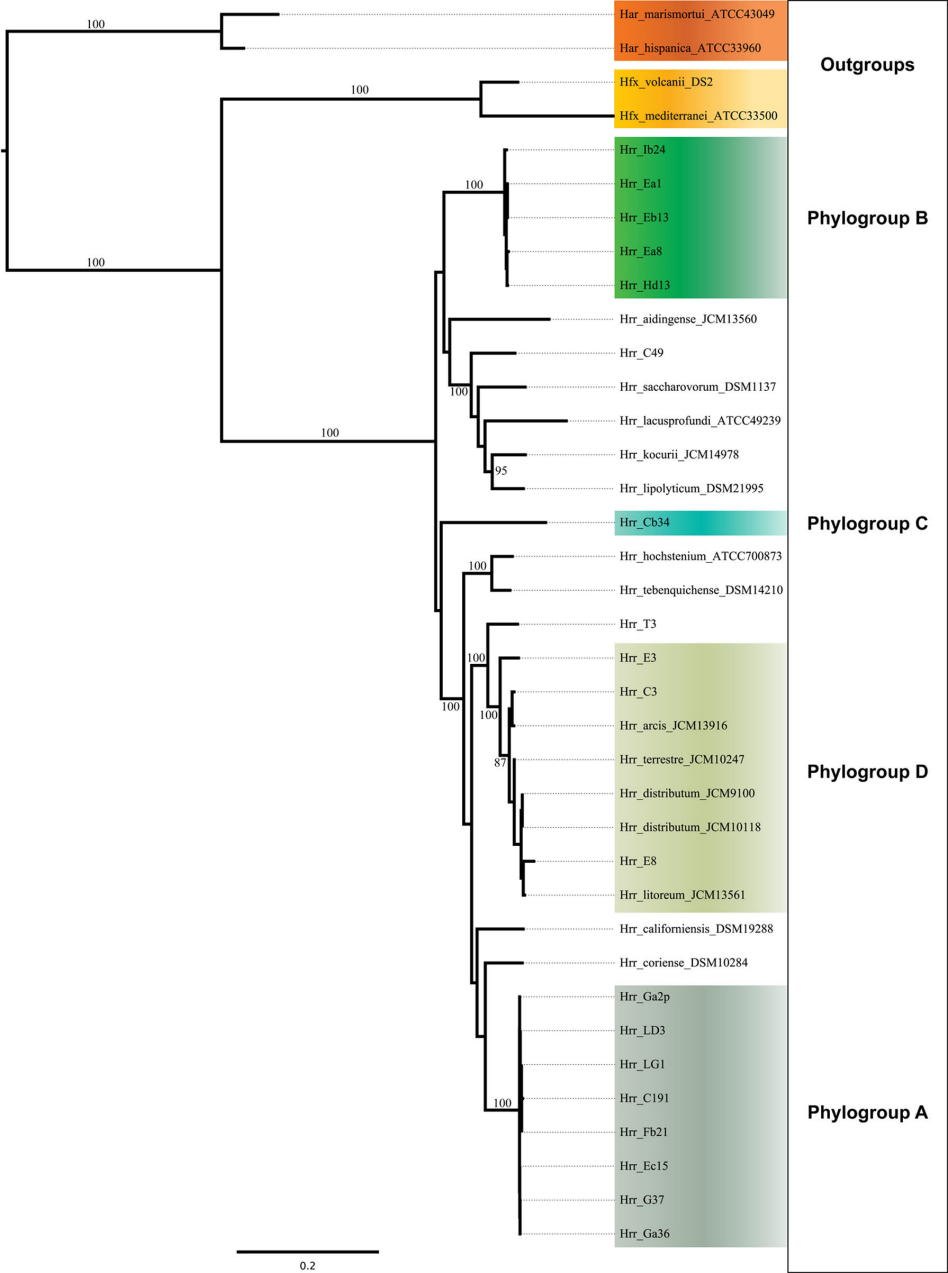
**Maximum-likelihood tree made from the concatenated DNA sequences of five housekeeping genes (*atpB, ef-2*, *glnA*, *ppsA*, and *rpoB*)**. Support values on branches are bootstrap replicates. Bootstraps values below 70 are not displayed.

Initial MLSA analysis (5-genes: *atpD*, *ef-2*, *glnA, radA, rpoB*) revealed the presence of three well-supported clusters [hereafter referred to as phylogroups *in sensu* (Papke et al., 2007)] within the canonical *Halorubrum* population of Aran-Bidgol (Figures 1, 2). A phylogroup was initially defined as a cluster of isolates with very low sequence divergence across the sequenced (MLSA) loci (<~1%). Seventeen of these isolates were then selected for genome sequencing for a higher resolution assessment. Selection criteria were biased toward the two larger phylogroups (A and B) to facilitate comparison between clusters. Only a single genome from phylogroup C was sequenced. Once genomic data were available, the PCR amplicons were replaced with the full-length genes from the assemblies. Further analysis made use of only these genomic sequences. The addition of the 19 NCBI genomes was made to provide context to the placement of the phylogroups within the genus and to determine their relationship with each other. The phylogenetic reconstruction including the type strains sequences revealed the presence of a fourth phylogroup (designated D) composed of three isolates from Aran-Bidgol and five type strains isolated from Central Asia and China (Figure 2).

### Phylogroups A and B are well-supported as discrete and cohesive entities

**Figure 3:**
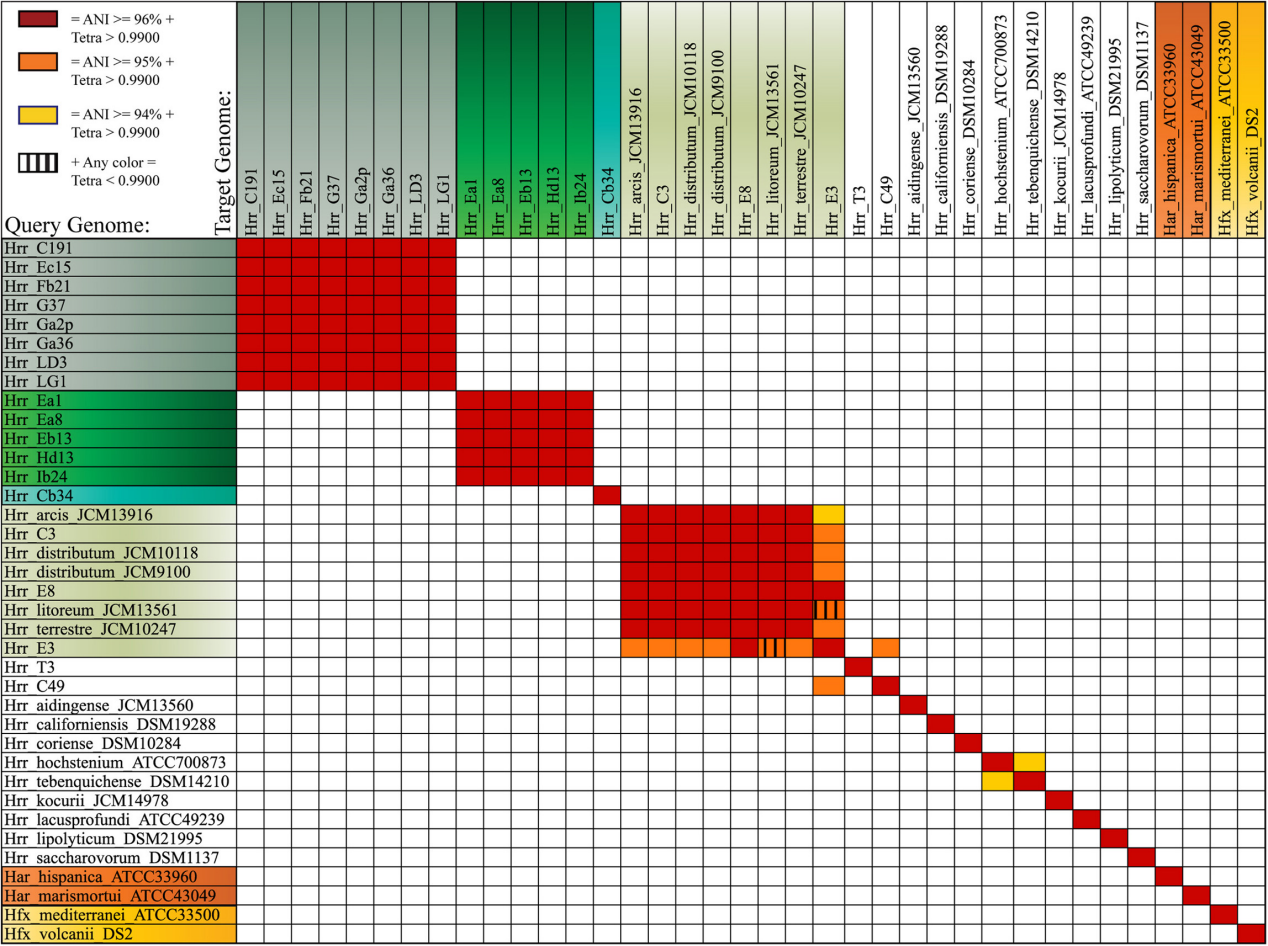
**Average Nucleotide Identity (ANI) and tetramer frequency correlation analysis**. Color coding reflects three described ANI cutoffs for species delineation. Red squares represent ANI values of 96% or greater, Orange 95% or greater, and yellow represents 94% or greater. The vertical stripes indicate tetramer regression coefficients lower than 0.9900.

**Figure 4:**
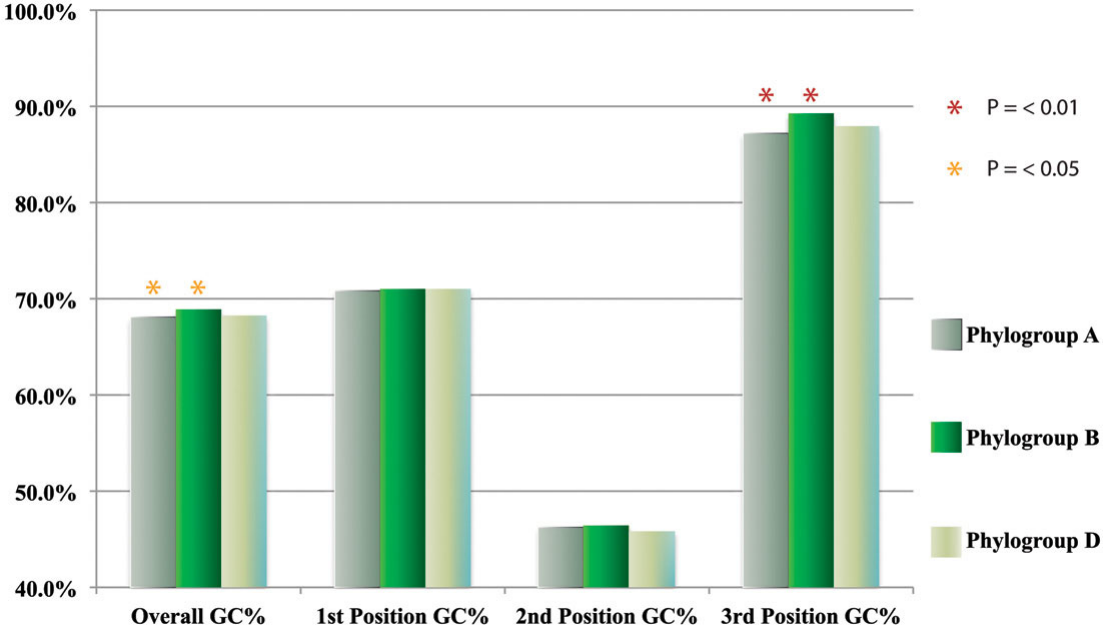
**GC usage of all annotated ORFs within and between phylogroups**.

The bootstrap values provided by the phylogenetic reconstruction strongly supported both phylogroups A and B. Individual gene trees and the concatenated gene tree returned support values of 99% or higher for all of the clusters (Figures 1, 2) and the trees showed no paraphyly with other taxa. Both phylogroups also displayed sequence divergence below 1% across the five loci (Table 5). Further, genome-level analysis (ANI) demonstrated similar results to the MLSA data (Figure 3). Additional support for these phylogroups came from the tetramer frequency analysis, which found no discordance amongst the members of either group, and each phylogroup displayed an intra-group ANI ≥98%. An analysis of G+C composition in the protein coding ORFs found that the strains within phylogroups A and B had a statistically different content in overall coding G+C and at the third codon position (*P* < 0.05 for both, Figure 4). Analyses of the inter-phylogroup differences showed the two phylogroups were quite different from each other and all other examined taxa. Both clusters were less than 97% similar in their pairwise MLSA distance to any other taxon in this study. Additionally phylogroups A and B were different from each other in tetramer frequency (below the 0.9900 correlation of Richter and Rosselló-Móra, 2009), ANI (only ~87% identity), and G+C content in the third codon position (*P* < 0.05; two-tailed *t*-test, Figure 4). Taken together these data support the notion that these phylogroups are discrete entities within a single environment, and that the individual phylogroups are cohesive.

**Table 5:**
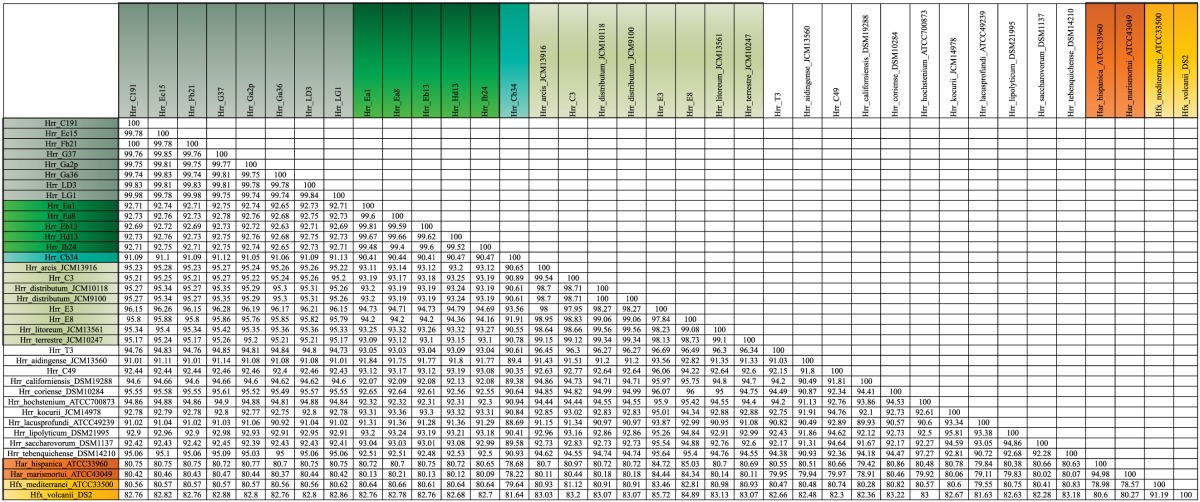
**Pairwise distances of the concatenated alignment of the five MLSA genes**.

**Figure 5:**
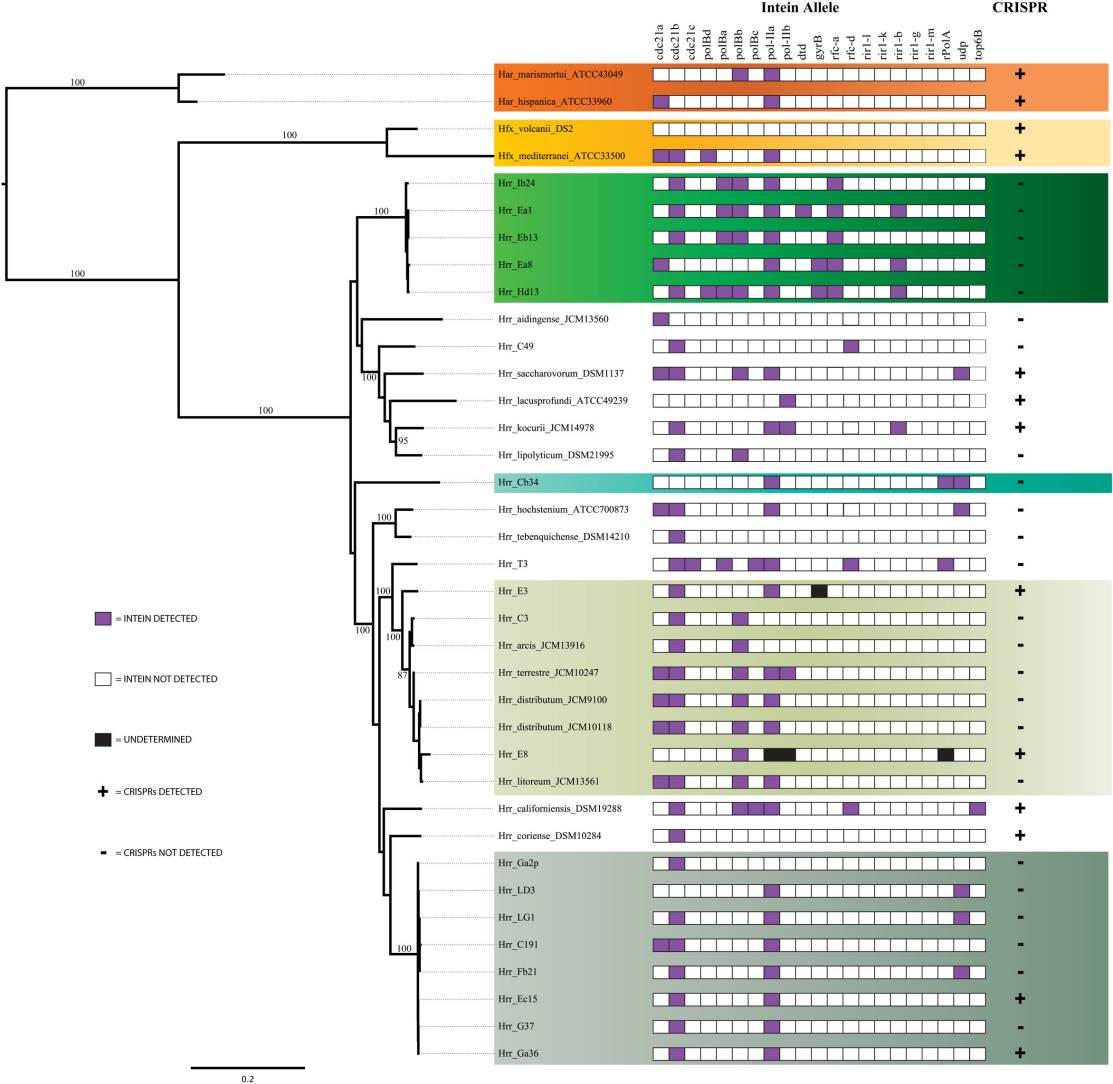
**Assessment of the presence of inteins and Clustered Regularly Interspaced Short Palindromic Repeats (CRISPRs)**. For inteins, purple boxes indicates the presence of an intein allele, white indicates its absence and black indicates an undetermined result. For CRISPRs a (+) indicates the presence of one or more CRISPRs and a (–) indicates the absence of CRISPRs.

To further evaluate the cohesion of the phylogroups a survey of inteins was performed. Inteins are molecular parasites that invade new hosts through horizontal transmission (Okuda et al., 2003; Swithers et al., 2013). Their patterns of presence and absence have been used as a barometer for horizontal transfer between closely and distantly related lineages (Swithers et al., 2013). Analysis of intein distributions supported earlier findings of cohesion within phylogroups and major distinctions between the phylogroups (Figure 5). Phylogroup A contains three non-fixed intein alleles that are present in more than half of the isolates, *cdc21*a, *cdc21*b, and *pol-II*a. Phylogroup B contains four non-fixed intein alleles also present in half or more of its isolates, *rir1-*b, *rfc-*a, *polB*a, and *polB*b but are absent from phylogroup A. Closer examination of the two shared alleles reveals that these inteins are not the same between the phylogroups. The *pol-II*a inteins in phylogroup B are 515aa long while those in phylogroup A are 494aa long, indicating an insertion or deletion event occurred in one of the phylogroups before the intein spread through the population. The preservation of the insertion or deletion within the phylogroups indicates that gene flow is occurring more readily within phylogroups than between, even when the same intein allele is shared. In accordance with earlier evidence, within phylogroups the intein sequence similarity is much higher than between phylogroups. It is unlikely that intein lengths are the result of sequencing or assembly artifacts, as they are constant within phylogroups.

**Figure 6:**
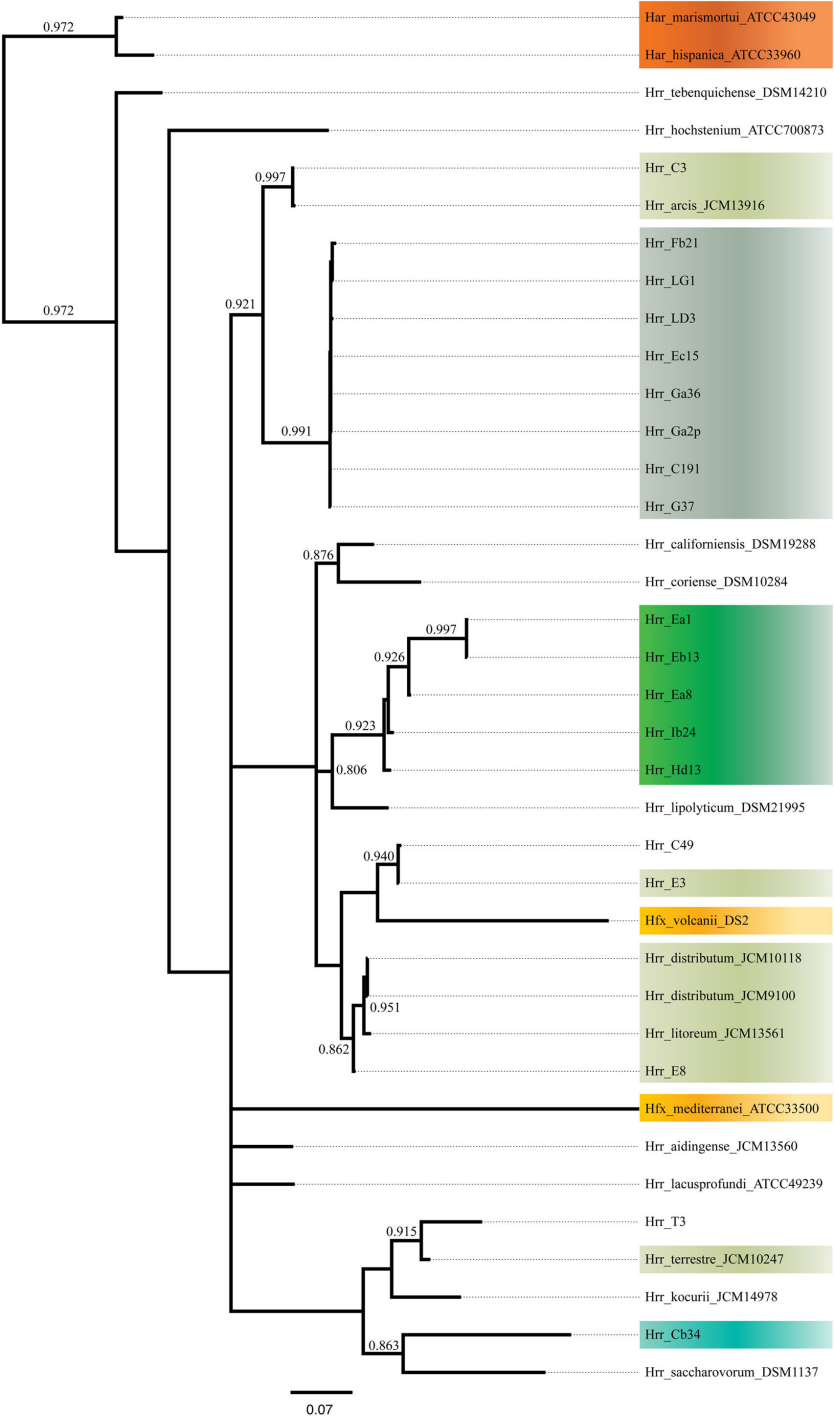
**Bayesian tree made from presence-absence of intein alleles and protein sequences of present alleles**. Support values on branches are posterior probabilities. Posteriors below 0.8 are not displayed.

The phylogenetic reconstruction derived from the combined presence-absence data and intein sequence data (Figure 6) shows clustering among phylogroup A and B of their constituent taxa. None of the taxa placed anywhere else but with the other members of its phylogroups and the posterior probabilities for these placements are high (0.991 for A and 0.923 for B). These results indicate that inteins are diverging mainly along cluster boundaries, as phylogroups A and B are distinct and separate, which further suggests that it is more challenging for the inteins to migrate outside compared to inside their phylogroups.

Another genetic element that serves to distinguish phylogroups A from B is the relative presence of CRISPRs. CRISPRs are a type of microbial innate immunity that provides a record of MGEs previously encountered by the lineage that carries them. This record serves the organism by recognizing and destroying sequences that resemble previously encountered MGEs. CRISPRs have been reported in 90% of surveyed archaeal genomes (Kunin et al., 2007), thus the presence and similarity of CRISPR loci provides a means for comparing the phylogroups. The distribution of CRISPRs was surprisingly patchy in phylogroup A and the genus as a whole; however, even more surprisingly was that putative CRIPSRs were absent in phylogroup B indicating its members may be devoid of them entirely (Figure 5). To assess if the absence of CRISPRs was an artifact of using draft genome assemblies, we tested for a correlation by relating N50 to CRISPR counts per genome and found there to be no correlation (*R*^2^ = 0.105, *P* > 0.05). Therefore, the CRISPR absences do not appear to be a result of genome assembly.

### Phylogroup D is not a cohesive and discreet entity

Phylogroup D appeared in the phylogenetic reconstructions of MLSA genes after the inclusion of the NCBI *Halorubrum* genomes. It includes five genomes representing four previously described *Halorubrum* species (*Hrr. arcis*, *Hrr. terrestre*, *Hrr. Distributum,* and *Hrr. litoreum*). It was surprising that multiple named species formed such a unit, but evidence suggests it is not discreet and cohesive like phylogroups A and B: much of the data conflict leading to an ambiguous demarcation of its boundary (see below).

The phylogenetic reconstruction of this cluster is supported by the bootstrap values, with exceptions. The concatenated phylogeny has a bootstrap value of 100 at its base and the individual gene trees each support the cluster with bootstrap value of greater than 80 (Figures 1, 2). Pairwise identity between the MLSA genes shows phylogroup D meets the initial criterion of <1% sequence divergence (Table 5). While high, the intra-cluster sequence identity is statistically lower than both phylogroup A and B values (*P* < 0.05, two-tailed *t*-test). ANI gives similar results to the pairwise identity (Figure 3): the intra-cluster value is ~97%. However some members of the group do not meet the 96% threshold identity, such as E3. Tetramer analysis shows good cohesion within the group, as all but one genome (E3) passed the cutoff. Both E3 and *Hrr. litoreum*'s tetramer frequency patterns are poorly correlated and are below the 0.99 coefficient cutoff advocated by the JSpecies 1.2.1 (Richter and Rosselló-Móra, 2009) package. As tetramer patterning is largely a granular filter, it strongly suggests that E3 and *Hrr. litoreum* may be distantly related, which is further supported by the ANI analysis.

The phylogroup D intein distribution patterns and sequences identities are dissimilar to phylogroup A and B (Figure 5). The intra-phylogroup identity of *pol-II*a is quite low in D compared to phylogroups A and B (~78 vs. ~99% and ~89%, respectively). The inter-group identities are much higher between B and D than in any other phylogroup relationship (~71%). These relationships are partly explained by *Hrr. terrestre*, which features an intein of much greater length and sequence divergence than the other alleles. This intein shares no more than 55% identity with any other phylogroup D *pol-II*a allele. If it is removed from consideration, the phylogroup D intra-cluster identity increases to ~99%. The relatedness to phylogroup A rises to ~53% while the value to phylogroup B is 76%. Intra-phylogroup D *cdc21b* diversity is nearly the same as its inter-phylogroup D diversity, which further indicates phylogroup D is a fuzzy entity. The intra-phylogroup identity for the *cdc21b* intein is ~91% (as compared to ~100% for A and ~99% for B) and its inter-phylogroup values are not much lower with D vs. B at ~83% and D vs. A at ~87%. However, the remaining taxa (*Hrr. arcis*, *Hrr. litoreum*, *Hrr. distributum*, *Hrr. terrrestre*, E8, and C3), including the named species appear to form a stable phylogroup. These data suggest that phylogroup D as constructed in our analysis is an amalgamation of populations that resembles other analyzed phylogroups but is not a cohesive unit upon additional investigation. The phylogenetic reconstruction derived from the combined presence-absence data and intein sequence data (Figure 6) shows that phylogroup D does not retain monophyly. Members place at four locations in the tree. The phylogroup displays high identities for core members, but “fringe” members are at the edge of inclusion.

*Hrr.* T3 and E3 presented significant challenges to defining the boundary of phylogroup D. As mentioned above, *Hrr.* T3 placed directly sister to the phylogroup in three of five gene phylogenies and inside the group in a fourth (Figure 1). In the fifth phylogeny it placed several nodes away from the cluster. The concatenation also places it sister to the cluster with maximum bootstrap support. However, its branch is long relative to the phylogroup. As noted, the pairwise identities and ANI values (Figure 3) both place it below the values seen inside the cluster. These notably lower values were used to exclude this taxon from the phylogroup. *Hrr.* E3 is less of a clean-cut case. Its *glnA* gene is outside of the phylogroup. It also falls on a branch by itself at the base of the cluster with rest of the phylogroup supported by an 87% bootstrap score. However, its intra-cluster pairwise and ANI values are several percent higher than *Hrr.* T3 and only a percent or two below most of the other members of the phylogroup. Overall, the ANI support was on the edge of current cutoffs for species delineation (95% or 96%) (Konstantinidis et al., 2006; Richter and Rosselló-Móra, 2009). Its genome had ANIs ~95% to most of the others in the phylogroup and was only 94% to *Hrr. arcis*. Further, E3's tetramer frequency was also substantially different from *Hrr. litoreum*. A possible explanation for some of these differences is that C49 and E3 show a high degree of sequence identity (95% ANI). It is also C49 with which E3's *glnA* gene associates. Finally, the combined presence-absence and intein phylogeny places these taxa together (Figure 6). These data suggest that the two lineages may have engaged in a recent round of genetic exchange, which might explain why E3 is on the periphery of phylogroup D. Ultimately, it was concluded to include E3 as a member of the phylogroup with the acceptance that it was probably an arbitrary distinction in either direction. It was this difficulty in defining the border that resulted in closer examination of phylogroup D and the ultimate rejection of it representing the same sort of entity that phylogroups A and B are.

## Discussion

### Are phylogroups species?

The data presented here raise the question: are phylogroups species? We use the term “phylogroup” because a polyphasic analysis (currently defined for the Halobacteria by Oren and Ventosa, 2013) for species description has yet been published on any of the clusters. Still, an evaluation of the data strongly suggests that at least some phylogroups will be eventually described as new species. From the phylogenetic data the perspective provided by the type strain sequences would indicate that phylogroups A and B are unique species. The ANI data support the idea of phylogroups A and B belonging to separate, novel species as several studies advocate cutoffs for species delineation (Konstantinidis and Tiedje, 2005; Konstantinidis et al., 2006; Richter and Rosselló-Móra, 2009) and phylogroups A and B meet all of them. Additionally, both phylogroups form a cohesive cluster with no particular affinity for other clusters, as evidenced by the strong bootstrap support at the base of each cluster. Also, phylogroups A and B are separated from the others by multiple type strains that place between them. Despite many of these branches being poorly supported, their placement and the strong cohesion within the phylogroups argue that the clusters indicate meaningful phylogenetic splits. These splits likely represent barriers that affect the frequency of gene flow between phylogroups, but not within.

Despite the phylogroups' seemingly species-like attributes, each gene analyzed demonstrates a different topological relationship for them, which means species cannot be viewed as a group of individuals that have a common ancestor, as would be expected from eukaryotic species. While the individual organisms in a prokaryotic species do not share a common ancestor, some of their genes will. For instance, analysis of marine *Vibrio* strains showed that ~1% of the genes within populations shared a common heritage (Shapiro et al., 2012), thus the term species in prokaryotes reflects a process of homogenization, but not heritage, the assumption of Darwinian tree-like speciation. A model that could explain the data is that genes are recombined frequently within *Halorubrum* populations and less so between them. Within the high frequency recombination background new genes that confer selective advantage constantly enter phylogroups from outside the population. These advantageous genes/alleles rise rapidly in frequency throughout the recombining population causing them to diverge in comparison to other phylogroups, yet remaining homogenized within. Like continental drift gives the appearance of discreet units yet are comprised of parts derived from other continents, so too are these two *Halorubrum* phylogroups.

Phylogroup D demonstrates further the model above, as recombination from outside the group is causing divergence, and disallowing a clean species prediction compared to phylogroups A or B. Therefore, phylogroups D is unlikely to be a single species because it is less cohesive in other measurements, which reflects that it contains several previously described species and also that it has engaged in numerous gene exchanges with not-to-distantly-related organisms. Alternatively, since species assignment is a pragmatic endeavor it could be argued from our data and analyses that phylogroup D is a single species with more genetic diversity than found in A and B. The ambiguous relationships of *Hrr.* T3 and E3 suggest there are different recombination partners available to the cluster members. Such differential exchange partners are key elements in microbial speciation (Papke and Gogarten, 2012) and it could be that T3 and E3 are in the process of speciation from the other members of D, but is incomplete. Tetramer frequency data, which has been demonstrated to convey phylogenetic information (Bohlin et al., 2008a,b) casts doubt on the phylogroup representing a single species. It is less stringent than ANI, being more inclusive with the clusters it forms at typical cutoff values (Richter and Rosselló-Móra, 2009). For this reason, when tetramer frequencies are in disagreement it is likely that the two sequences being compared are not closely related. Thus, the tetramer frequency difference between E3 and *Hrr. litoreum* is also strong evidence for those two taxa not belonging to the same species. Interestingly, if T3 and E3 belong to different species and are removed from consideration, the remaining members of phylogroup D would be a single species by all measurements and cutoffs, and yet are still comprised of four named species. However, these strains were isolated from three different geographic regions of Asia at three different time points (Zvyagintseva and Tarasov, 1987; Ventosa et al., 2004; Cui et al., 2007; Xu et al., 2007), from Chinese solar salterns to Turkmenistani saline soils. While the role of geography and ecology in haloarchaeal speciation is unsettled (Oh et al., 2010; DeMaere et al., 2013; Dillon et al., 2013; Zhaxybayeva et al., 2013) all four of the named species have undergone polyphasic characterization, including DNA-DNA hybridization (Ventosa et al., 2004; Cui et al., 2007; Xu et al., 2007). Presumably, if these taxa lived in the same environments and exchanged genes with each other in a positively biased manner like phylogroups A and B, they would be homogenized and indistinguishable by current polyphasic description processes. What sets phylogroup D apart in our analysis is that we do not have population data on members from the same site, and cannot compare equivalently: if we had more data from natural populations like we do for phylogroups A and B, it might be possible to detect reliable differences that separate the named species into different MLSA phylogroups. For example, dozens of *Sulfolobus* strains isolated from geographically distant sites were less than 1% divergent across multiple loci, yet population data analysis demonstrated they fall into discreet clusters associated with geography (Whitaker et al., 2003) While the taxonomy of the Halobacteria is in flux (for example: McGenity and Grant, 1995; Oren and Ventosa, 1996) it seems unlikely that these four separate species will be merged into one. Recent work has served to split *Hrr. terrestre* from *Hrr. distributum* (Ventosa et al., 2004). Thus, it is challenging to conceive of phylogroup D as a single species, which serves as a strong example of the limits to MLSA and ANI in regards to being the defining measurements of species.

### CRISPR distribution may be the result of selection

It is important to acknowledge that the patchy CRISPR distribution may be in part an artifact of genome assembly. Repeats can prove a challenge to assembly of short read data (Miller et al., 2010; Magoc et al., 2013) and CRISPRs are repeat heavy. However, false negatives that may exist are unlikely to be directly correlated with assembly quality, and no significant correlation is found between N50 score and the number of CRISPR arrays detected (*P* > 0.05). Additionally, the use of a different CRISPR detector, Crass v0.3.6 (Skennerton et al., 2013), which analyzes raw sequencing reads, rather than finding them in assemblies, supported the CRISPRs reported and found only slight evidence for three additional taxa possessing CRISPRs (data not shown). This would only represent individual CRISPR repeats no larger than about three spacers. While CRISPRs this size have been reported (Kunin et al., 2007) the evidence is inconclusive and if these three taxa do possess CRISPRs their distribution would remain sparse. Only seven of the 18 genomes sequenced in this study would possess them.

CRISPRs have been reported to be very common in the archaea (Jansen et al., 2002; Godde and Bickerton, 2006; Kunin et al., 2007; Held et al., 2010) with reported incidence as high as 90% (Koonin and Makarova, 2009). The incidence in bacteria is closer to 50%. The higher incidence in the archaea may be due to the underrepresentation of archaeal genomes in databases. With viruses and other MGEs so common (for discussion of haloviruses see Dyall-Smith et al., 2003; Porter et al., 2007) and horizontal transfer of CRISPRs a frequent occurrence (Kunin et al., 2007; Sorek et al., 2008), why does selection ever conjure a no-CRISPR lineage? One possibility is that the benefit provided is not strong enough to outweigh the costs, as CRISPR systems require precise matches with their target, and a “proto-spacer” with one or two mismatches can eliminate functionality (Deveau et al., 2008). The loss of cassettes in CRISPR arrays is not uncommon (Deveau et al., 2008; Díez-Villaseñor et al., 2010; Touchon and Rocha, 2010), while loss of an entire array is less so (Held et al., 2010; Touchon and Rocha, 2010). Possession of large CRISPR arrays may not offer extra protection against the viruses in an environment (Díez-Villaseñor et al., 2010). It might be that if predation level by MGEs rise and fall then the value of the CRISPR system might follow those trends. *Escherichia* and *Salmonella* CRISPR arrays do not appear to deteriorate rapidly enough to be lost entirely and they show a high rate of transfer and loss of the *cas* proteins that form the machinery of the functional system (Touchon and Rocha, 2010). This might suggest that the need for the system may not be constant. Another reason for degradation of the system could be related to it behaving in an auto-immune fashion. When challenged by artificial constructs including a proto-spacer and a gene complementing an autotrophic defect in the strain, *Sulfolobus* cells developed a surprisingly large number of deletion mutants in the spacer providing immunity to the construct (Gudbergsdottir et al., 2011). The authors speculated that there might be some small degree of feedback where the system attacks the host's spacer in addition to that of the MGE. The cellular repair systems may then easily delete the spacer during the repair process. Feedback against self and similar to self DNA, such as targeting closely related housekeeping genes (Gophna and Brodt, 2012) could also impact mating proficiency if the CRISPR system degrades the DNA of exchange partners before it can experience recombination events. It is also important to consider that mechanisms other than CRISPRs have major roles in developing resistance to MGEs (Wilson and Murray, 1991; Bickle and Krüger, 1993; Díez-Villaseñor et al., 2010). For instance, there could be a balance between CRISPRs and restriction/modification systems where one system is lost and another replaces, or complements it such that any one anti-MGE mechanism at any moment in time is in flux.

### The absence of inteins suggests barriers to recombination between phylogroups

Inteins are found pervasively among the archaea (Perler, 2002). They insert into genes and once translated their splicing domains use an auto-catalytic mechanism to self-excise from the protein and re-join the two halves of the polypeptide to generate a functional protein. Inteins associate with homing endonucleases (HEN), found between the splicing domains, to allow their transmission into new hosts. HENs target highly conserved sites in highly conserved genes (Swithers et al., 2009). These HENs appear to be extremely specific in their target sequences as inteins are only found inserted among the most conserved residues of highly conserved protein coding genes (Swithers et al., 2009). Their means of dissemination from host to host is, as yet, unknown although it is clear that it relies on established methods of gene flow within a population (Goddard and Burt, 1999; Gogarten and Hilario, 2006). This suggests that if two hosts have no method of transmitting genes between themselves then the resident inteins will not cross hosts, either. Thus, the patchy distribution of inteins can be interpreted as evidence for a barrier to transfer. This is particularly relevant for the alleles that are not shared between phylogroups A and B. The presence of multiple alleles not seen in the other group argues that the allele has been unable to spread. This is not implying that members of phylogroups A and B do not exchange genes, rather, the sequence divergence and lack of intein spread implies that the recombination process is hindered relative to within group genetic exchange. Indeed, if the mating observed between different *Haloferax* species (see Naor et al., 2012) is possible then almost any sequence divergence between *Halorubrum* phylogroups is akin to a speed bump rather than a mountain in slowing the rate of genetic exchange. Additionally, studies of homologous recombination have found transfers across class-level phylogenetic distance, only at increasingly lower rates as the genetic distance increases (Vulić et al., 1997; Williams et al., 2012).

## Author contributions

Matthew S. Fullmer, J. Peter Gogarten, Antonio Ventosa, and R. Thane Papke participated in the design of this study and helped to draft the manuscript. Shannon M. Soucy generated the intein data and performed the majority of the intein analysis and helped to draft the manuscript. Kristen S. Swithers performed the CRT analysis and helped to draft the manuscript. Andrea M. Makkay and Ryan Wheeler performed the MLSA PCR. Andrea M. Makkay performed the genome sequencing. All authors read and approved the final manuscript.

### Conflict of interest statement

The authors declare that the research was conducted in the absence of any commercial or financial relationships that could be construed as a potential conflict of interest.
